# Animal assisted interventions in the children's hospital: protocol for a scoping review

**DOI:** 10.12688/hrbopenres.13143.2

**Published:** 2021-05-14

**Authors:** Rachel Howe, Sandra Nicholson, Attracta Lafferty, Carmel Davies, Diarmuid Stokes, Thilo Kroll

**Affiliations:** 1 School of Nursing, Midwifery & Health Systems, University College Dublin, Donnybrook, Dublin 4, D04 VIW8, Ireland; 2 School of Veterinary Medicine, University College Dublin, Donnybrook, Dublin 4, D04 W6F6, Ireland

**Keywords:** animal assisted activities, pet therapy, paediatrics, children's hospital, scoping review protocol

## Abstract

The introduction of Animal Assisted Interventions (AAIs) in healthcare is relatively common; however, their actual effectiveness and long-term impact are not so well known, especially in relation to the children’s hospital setting.  It is important to plot where and why animal interventions take place but also to focus on how the human animal bond impacts on children in a children’s hospital setting.  Family members, including companion animals, are important supports which help children to relax and give them a sense of familiarity to navigate the busy and stressful hospital environment.  The scoping review of the literature proposed will explore the scientific evidence base for AAIs in children’s hospitals and will map results prior to undertaking a full scale research project.   Arksey and O’Malley’s framework guided by the Joanna Briggs Institute will frame this review protocol.  Appendices are used to ensure transparency of methods. The protocol is presented in narrative style to demonstrate flow, fluency, and appeal to wider readership.

## Background

Children and young people hospitalised for surgery or medical treatment can find the experience as a most distressing event in their lives if not managed appropriately by healthcare professionals and their families ( Bjork *et al.*, 2005; Coyne, 2006; Darbyshire, 1994; Gariépy & Howe, 2003; Gibson *et al.,* 2010). Traditionally, anxiety was managed largely through pharmacological treatments resulting in negative consequences in terms of side effects of medications and ongoing costs or demands on the health care service. Non-pharmacological interventions including play and distraction therapies have positive effects on a child’s recovery and general management of care in hospital ( Al-Yateem *et al.,* 2016; Gariépy & Howe, 2003). Gibson *et al*. (2010) identified that all child participants in their study reported some dislike of the hospital environment. Young children in hospital requested toys from home and older children found some comfort in photographs of pets as well as soft furnishings including their own pillows and bed covers helped in improving the hospital environment to be more homely ( Gariépy & Howe, 2003; Gibson, *et al.*, 2010; Lambert, *et al.,* 2014). Coyne’s qualitative study; of 11 children aged seven to 14 years, located in four paediatric units in the United Kingdom; reported children missing aspects of their home life specifically, “ *Miss my mum, my dog, my sister, the atmosphere, my own bed*” ( Coyne, 2006 pp. 329). Family members, including family pets, are important supports which help children to relax and give them a sense of familiarity to navigate the busy and stressful hospital environment.

The hospital environment while daunting and sometimes considered ‘ *a scary place*’ for children, it is also a busy and stressful place for the children’s parents, siblings and healthcare staff working each day in what is considered an under resourced environment ( Bjork *et al.*, 2005; Bridgeman *et al.*, 2018; Gibson *et al.*, 2010). Families often experience chaos and extreme loneliness throughout episodes of hospitalisation and their lives are broken, similar to, “ *an earthenware pot dropped and broken into pieces*” ( Bjork *et al.*, 2005 pp. 270). The outcome of Gariépy & Howe’s (2003) mixed methods research study comparing children attending a hospital based treatment clinic and a healthy cohort of children in a crèche found that children need to be provided with facilitated time to access toys and activities on a regular basis to help them process their anxiety and stress caused by their illness, treatment and ongoing health concerns. Gariépy & Howe (2003) suggested future research involving observation and controlled intervention would be of benefit, particularly on an individual basis, so that the child could develop a trusting relationship with the researcher and work through which intervention best suits their needs according to their age and stage of development.

Consideration should be given to other interventions such as innovative non-pharmacological interventions or complementary therapies which include Animal Assisted Interventions (AAIs). There is ambiguity in the international literature in relation to the definition and adaptation of the terms AAI (which is the umbrella term) animal assisted activities (AAA) and animal assisted therapy (AAT). Some studies use either concept or use them interchangeably. The subsequent scoping review will provide a clear definition of these terms from the literature. The following definition of AAI from The International Association of Human-Animal Interactions Organizations ( IAHAIO, 2018 p.5) has been adopted by the review team:

*‘’An Animal Assisted Intervention is a goal oriented and structured intervention that intentionally includes or incorporates animals in health, education and human services (e.g., social work) for the purpose of therapeutic gains in humans. It involves people with knowledge of the people and animals involved. Animal assisted interventions incorporate human-animal teams in formal human services such as Animal Assisted Therapy (AAT), Animal Assisted Education (AAE) or under certain conditions Animal Assisted Activity (AAA). Such interventions should be developed and implemented using an interdisciplinary approach.’’*

 In AAIs, the animals themselves are the health intervention. An initial search of the literature showed that AAIs enhance positive feelings in people, raise oxytocin levels, promote improved mood and foster trusting relationships ( Fine, 2015). A recent protocol and meta-analysis by May *et al.* (2020) aimed to quantify the impact of brief canine therapy within the discipline of psychology. Significant positive impact of animal assisted therapy (AAT) on subjective anxiety of hospitalised children was reported by May *et al.* (2020). While this systematic review and meta-analysis was undertaken within the paediatric context it focused primarily on the psychological and physiological responses of children, reporting the impact of canine therapy on outcomes associated with pain and anxiety. Studies in the review were limited to randomised controlled designs ( May *et al.* 2020). Further investment into the scientific literature of this safe, novel and efficient AAT was recommended by May *et al.* (2020). The proposed scoping review is broader in its approach and will include all study designs and all types of AAIs, not only canine therapy. 

A preliminary search of the international literature completed in early 2020 revealed the following studies. A critical review by an Australian team Chur-Hansen *et al.* (2014) found only nine studies worthy of inclusion in their review. The authors reported, ‘ *methodological challenges*’, in their review and indicated the need for future research to be more rigorous, to add to the evidence base in this area of AAI (Chur-Hansen *et al*. pp. 5). Since then, Vagnoli *et al*. (2015) investigated the effectiveness of AAI as a distraction for reducing Italian children’s pain and distress during and after phlebotomy. Significant differences were found between the intervention and control group for example serum cortisol levels were significantly lower denoting less stress/anxiety. Limitations in this trial included the small sample size of each group. Children who participated were aged four to 11 years and were not previous dog owners. In future research, consideration could be given to child participants, choosing whether or not they want to participate in the selection of an appropriate comparison intervention based on child participatory research methods.

Calcaterra *et al.* (2015) explored the post-operative benefits of AAT on children’s stress and pain in the acute children’s hospital setting in Germany. Data collection involved recording of baseline parameters, salivary cortisol levels, electroencephalogram and an assessment of pain. No significant difference were reported in cortisol levels and the authors recommended alternative hormone level measures be considered in future studies. Future studies should consider more comprehensive monitoring of hormone levels as well as cortisol levels and recruit a larger sample.

A key piece of literature from America by Hinic *et al*. (2019) evaluated the effect of a brief pet therapy visit and an active comparison intervention (jigsaw puzzle) on anxiety in hospitalised children. Findings support the AAI in reducing anxiety for children and their parents. Again a small study but with a comparison intervention. More rigorous studies of psychological and physiological outcomes both in the immediate and long-term phases were advised. The value of exploring more qualitative elements over a longer timeframe may also be worthy of study. 

The Royal College of Nursing (RCN) in the United Kingdom (UK) have published guidance for Health Care settings introducing therapy dogs ( RCN, 2018). An evaluation of an AAI service established in 2012 in one children’s hospital from UK demonstrated extremely positive results. Parents, staff members and children (n=200) surveyed reported the service to be ‘ *very worthwhile*’ (98%) ( Uglow, 2019 p.512). The benefits of AAI largely outweigh the risks and with relevant protocols in place the initiative should be repeated in other hospitals ( Uglow, 2019).

Dalton *et al*. (2020) and Gerardi *et al*. (2018) highlight the need for a One Health approach to ensuring minimal risk to human and animal participants in AAI programmes. It is very important to minimise risks such as bites and cross infection in the hospital context especially with vulnerable children. SN the veterinarian expert will advise the research team in relation to guidelines and policy required prior to implementation phase of the study. In Ireland anecdotal evidence points to pet therapy taking place in hospices, nursing homes, general hospital Intensive Care Units (ICUs) and one children’s hospital. Peata and Irish Therapy Dogs, the Irish charities, have pet visitation programmes in place to many healthcare settings which are currently stalled due to coronavirus disease 2019 (COVID-19) ( Peata, 2020).

Interesting themes meriting further exploration are emerging from the literature. A need exists for additional research studies that are well planned and follow a more definitive protocol have been suggested. Our review will map the existing literature available through peer-reviewed journaals irrespective of the quality. We also want to probe the literature to determine; Why has AAI been used? Where? And how effective was it? Were there implementation considerations? Were there particular research methods utilised and recommendations for future research?

A scoping review may be useful for informing the design of such projects. A scoping review would enable a deeper exploration of the scientific literature on how AAI might contribute to the child’s wellbeing during and after hospitalisation. It may also reveal some risks associated with AAI that may act as barriers to implementation. Any theories about the human-animal bond which the researchers might discuss in their articles can be mapped alongside other contextual data such as geographical location and types of animal interventions. The six stages in the Scoping Review Framework introduced by Arksey & O’Malley (2005) will help guide the review protocol and subsequent scoping review.

## Aims/objectives

The aims of the systematic scoping review are:

- To explore what is known about the scientific evidence base for AAIs in terms of their benefits for children in hospitals?
- To summarise and map the evidence on AAI since its conception and to determine where and when the research was carried out?
- To identify any gaps in the literature or designs worthy of further consideration and focus for future research.

## Stage 1. Identification of the Scoping Review Research Question

The population, concept and context (PCCo) framework was utilised to help form the research question for this scoping review which is supported by Anderson *et al.* (2008) and the Joanna Briggs Institute ( Peters *et al.*, 2017; Peters *et al.*, 2020):

*“What is known about the scientific evidence base for animal assisted intervention (C) with children and young people (P) in the children’s hospital (Co) setting?”*

## Stage 2. Identifying relevant studies

For a systematic review, it is important that as many relevant studies are sourced, rather than all studies ( Craven & Levay, 2019). The review question has been developed using the PCCo framework, so that the search terms can closely match the themes within the literature, to retrieve focused relevant studies, rather than all studies on the topic. Including all literature from date of conception on the topic of AAI in children’s hospitals will reduce the risk of bias ( Craven & Levay, 2019). Seminal works on human-animal studies stem from the 1980’s which was also the same time the first journal devoted to human-animal studies - Anthrozoos was published (DeMello, 2012). Therefore, it makes sense to limit the search to include literature published within the past 40 years.

### Inclusion and exclusion criteria

No exclusion for language will be applied in the initial search, since it will be important to map the sources and origins of the literature, to reveal any gaps in the evidence internationally. English published studies tend to be biased towards positive findings compared to non-English-language studies where results may contain null or negative findings according to Boland *et al.* (2017). The review team possess international language skills making the translation of research titles and abstracts of non-English studies possible. Rasmussen & Montgomery (2018) warn that ignoring non-English studies risks ignoring key data. All results will be charted regardless of language and if funding is available and the article is considered to be a valuable addition to the review then translation of the full article may be possible. The review team believe that evidence should be more accessible globally as there are over 9,000 peer-reviewed journals published in other languages ( Curry & Lillis, 2018).

Initially it was envisaged that the search terms entered, regarding context, would need to be restricted to the acute children’s hospital setting. This exclusion was considered necessary to ensure the focus of the review question is answered and limit the retrieval of irrelevant information outside of the acute children’s hospital setting. However, following an initial search of two to three databases there was a paucity of results and the search term for context was then expanded to ‘hospital’ rather than ‘acute children’s hospital.’ A broad search for data is consistent with scoping reviews ( Arksey & O’Malley, 2005; Peters *et al.*, 2017).

### Types of studies

Primary studies of any study design will be eligible for inclusion. In addition systematic reviews will be considered. Opinion pieces, commentaries and editorials that do not present data meeting the inclusion criteria will be excluded. Purely, theoretical discussions will be excluded. It is necessary to ensure a broad search initially including all data and reviews so that it can be mapped, with any gaps identified ( Tricco *et al.*, 2016). 

### Types of populations

Children and young people in the children’s hospital setting is the focus of this review and therefore the adult population will be omitted from the search terms. AAI has been researched in other populations so it will not be necessary to include them in this review. The rationale for this exclusion is important so that information can be retrieved within the focus of the review question which is AAIs in children’s hospitals. The definition of a child is a person below the age of 18 years old which is adapted from the World Health Organisation ( WHO, 2014) and United Nations. Animal Assisted Interventions may not be appropriate for infants or children with an aversion to animals, however all instances of AAI will be mapped from the search results. No restriction on the type of animal intervention will be installed if it meets the population criteria of being carried out in a children’s hospital.

### Types of interventions or exposure

The search string for the concept relating to animal assisted interventions will also include sibling terms such as; animal assisted activity (AAA), animal assisted therapy (AAT), animal assisted coaching (AAC) and animal assisted play therapy (AAPT). Keywords and search strings for each concept and their associated terms are listed in Table 1. The MeSH thesaurus tool within Pubmed will be utilised and thesaurus results shared across each database search to maximise search results as advocated by Sampson *et al.* (2009). Literature citing all types of animals will be included in the initial screening of results so that the type of animal involved in the interventions can be charted, coded and cross checked throughout the entire data set for comprehensive reporting of results. 

**Table 1.** Keywords for each search string.

1. **Population (P):** child or child, pre-school, minors, children or adult children or children, adult or young person or young people or paediatric or paediatrics or pediatric or pediatrics or toddler or pre-schooler or “adolescent” [Mesh] = adolescents or adolescence or teens or teen or teenagers or teenager or youth or youths or adolescents, female or adolescent, female or female adolescent or female adolescents or adolescents, male or adolescent, male, or male adolescent or male adolescents
2. **Concept (C):** animal assisted interventions or interventions, animal assisted or animal assisted activity or animal assisted activities or activity, animal assisted or activities, animal assisted or animal assisted coaching or coaching, animal assisted or animal assisted play therapy or play therapy, animal assisted or “animal assisted therapy” [Mesh] = animal assisted therapies or assisted therapies, animal or assisted therapy, animal or therapies, animal assisted or therapy, animal assisted or animal facilitated therapy or animal facilitated therapies or facilitated therapies, animal or facilitated therapy, animal or therapies, animal facilitated or therapy, animal facilitated or pet therapy or pet therapies or therapies, pet or therapy, pet or pet facilitated therapy or facilitated therapies, pet or facilitated therapy, pet or pet facilitated therapies or therapies, pet facilitated or therapy, pet facilitated
3. **Context (Co):** children* hospital or hospital, children* or “child, hospitalized” [Mesh] = children, hospitalized or hospitalized children or hospitalized child or child, hospitalised or children, hospitalised or hospitalised children or hospitalised child or “hospitals, pediatric” [Mesh] = pediatric hospitals or hospital, pediatric or pediatric hospital or hospitals, paediatric or paediatric hospitals or hospital, paediatric or paediatric hospital or hospital ward, or children’s ward or paediatric ward or pediatric ward or children’s unit or paediatric unit or pediatric unit or children’s clinic or paediatric clinic or pediatric clinic
Results from 1. and 2. will be combined and then further combined with 3. to reveal full list of articles to be initially screened by title and abstract

### Setting/ context

It is important to search broadly yet comprehensively for literature pertaining to AAI in the children’s hospital as it is the phenomenon of interest to the author’s work. However, it is likely that other settings and contexts will be found following the initial search to ensure comprehensive search results are obtained. Irrelevant citations will be excluded by reviewers following further screening and in accordance with exclusion criteria ( Table 3).

### Search strategy

In order to produce a comprehensive map for the scoping review the following databases will be searched:

*Health databases:* PubMed, CINAHL Plus and the Cochrane Library.

*Social Sciences databases:* PsycINFO, Applied Social Sciences Index and Abstracts (ASSIA) and the Social Sciences Citation Index.

*Veterinary Medicine:* CABI VetMed Resource and Ingenta Connect

The need to search more than one bibliographic database is justified since the research question crosses more than one discipline and a broad search of the literature is required for the review ( Boland *et al.*, 2017). A decision was made by the research team not to search the grey literature for the purposes of this review, but to focus on the published literature in an effort to ensure a higher scientific value of research is searched and reported.

The keywords; animal assisted therapy; child, hospitali*ed; adolescent; paediatrics and hospitals, paediatric; will be entered into the search field individually then combined for each of the database searches having been carefully selected from the review question and their associated search strings Table 1.

The Joanna Briggs Institute advocates the invaluable skills of a research librarian for refining search terms and utilising tools to their maximum capacity ( Peters *et al.*, 2017 & Peters *et al.*, 2020). The author has access to a librarian (DS) with expert experience in carrying out searches using thesaurus tools and will provide assistance and support to the researcher carrying out the database searches. The highly sensitive search strategy using boolean operators, both free-text and subject headings (including MeSH when available) for each database search will be reported to ensure transparency through a clear audit trail.

Booth (2008) asserts the importance of reviewers utilising a range of techniques from their ‘ *toolbox*.’ More traditional methods such as citation searching, contacting authors and web searching will also be employed to enhance efforts to produce a comprehensive review of published information. Hand searching for journals may reveal additional information, because not all reports are indexed correctly and could be missed without searching manually. Citations from articles included in the full text review stage will also be obtained if not already included in the initial search results.

## Stage 3. Study selection

Each search will be saved and imported into a file on Endnote X9. The advantage of Endnote is that a research diary of each search can be made by dating each file upon import of search results and the number of articles retrieved can then be added to the Preferred Reporting Items for Systematic Reviews and Meta-Analyses (PRISMA) flowchart advocated by the Joanna Briggs Institute ( Peters *et al.*, 2017 & Peters *et al.*, 2020). A PRISMA flowchart will be produced following the completed searches which will enable transparency of reporting, demonstrate how decisions were made about excluding citations and allow for easy replication and comparison of any future searches.

Citations stored on Endnote will be imported to Covidence for ease of screening by the review team (RH and TK). Rationale for exclusions will be clearly tracked and logged by Covidence. Any disagreements can be quickly resolved by the third researcher (SN).

There is debate in the literature about the need for more than one researcher to undertake the initial screening of the citation titles and abstracts from the long list of search results ( Levac *et al.*, 2010; van den Berg *et al.*, 2013). Since a scoping review retrieves, “ *all relevant data regardless of study design*,” potentially thousands of results could be obtained ( Arksey & O’Malley, 2005 pp.20). Two reviewers (RH & TK) will double screen search results for inclusion based on title and abstract in line with other reviews published.

## Stage 4. Data charting

The second stage of screening results will be carried out independently by two researchers (RH & TK) using a reporting checklist or data charting form as it is known by for scoping reviews ( Levac *et al.*, 2010; Peters *et al.*, 2017). A sample of ten studies charted independently by both reviewers will be compared and discussed to pilot the tool and assess if results are consistent with the research question. This approach was advocated by Daudt *et al.* (2013) to improve charting of results so that the review question can be answered appropriately. Citation titles and abstracts included in the second screening will be full text results which will be shared with each reviewer through Covidence. This strategy has been reported in the literature as being an effective method ( Daudt *et al.*, 2013). A third reviewer (SN) will be available to discuss any differences or ambiguities in results. A sample data charting form; adapted from the Cochrane collection template and informed by Nicholson *et al.* (2019); is attached as Table 2. Since the process of scoping reviews is an iterative process so too will be the need to discuss the suitability of the data charting form once screening has commenced and is discussed by the review team. Any changes to the form will be noted in the researcher’s research diary along with any decisions about screening as a result of any meetings, reflections and actions taken ( Boland *et al.*, 2017; Daudt *et al*., 2013).

**Table 2.** Data charting form.

Report ID: #	Source Type (i.e. Journal article):
**General Information**	
Article Title	
Authors	
Country of origin	
Discipline (i.e. Med., Nurs., Psych., Vet Med.):	
**Introduction**	
Aims and Rationale	
Background Details	
Research Question	
**Participant Details**	
Sample Size	
Age	
Gender	
**Intervention Details**	
Type of Animal	
Animal Handler	
Comparison Intervention/ control (if applicable)	
**Environment**	
Study setting	
Specific disease group or condition (if specified)	
Rationale for intervention (i.e. pain/anxiety)	
**Methods** (if applicable)	
Sampling Strategy	
Study Design	
Theory/Framework	
Data Collection	
Data Analysis	
**Outcomes/** **Recommendations**	

Follow-up Required Yes No Contact details:

**Table 3.** Inclusion and Exclusion criteria for literature search.

Include	Exclude
All child 0–18 years	Adults
Inpatients	Outpatients
Hospital	Community
All languages	None
Peer reviewed	Non peer reviewed
Evidence Based Practice	Books
Journal articles	Conference proceedings
Dissertations	Media
Any methods	None
Reviews	Opinion pieces
Animals	Commentaries
	Editorials
	Pure theoretical discussions

## Stage 5. Collating, summarising and reporting the results

Each data charting form will be given a unique number to aid identification and discussion across the review team as advised by Daudt *et al.* (2013). A description and summary of the review findings will be the form of analytical interpretation of the review results which will be enhanced through discussion with the review team and supervisor to draw out meaningful results ( Vijayamohan, 2015). Peters *et al.* (2020) are definitive in stating that, “ *qualitative content analysis in scoping reviews is generally descriptive in nature*” and therefore this review protocol will be in keeping with the Joanna Briggs Institute most recent publication. The use of innovative charting tools such as word clouds will be considered to visually represent definitions of animal assisted activities as an example. Other charted results will require more traditional narrative analysis as discussed by Levac *et al.* (2010).

### Assessment of methodological quality

Traditionally the appraisal of methodological quality and risk of bias of the included articles are not consistent within the conduct of a scoping review ( Levac *et al.*, 2010). Therefore, the methods of each individual article will be charted only and the researcher will report the methodologies utilised throughout the discussion and synthesis of findings in the final report of the review.

## Stage 6. Consultation

Consultation for this scoping review will be in the form of a reference panel to support the reviewers with the review. Networking with the Authors’ University and School of Veterinary Medicine has already yielded excellent sources of information on animal welfare which will be an important concept to guard later in the research study. One member of the research team (SN) has expertise in the area of animal welfare and will be key to guiding and ensuring that the welfare of animals as well as research participants will be considered throughout the project. This review is part of a larger study and the scoping review will feed into the design of a primary research study. Any consultation with children will require ethical approval and this will be obtained before any consultation takes place later in the research project.

### Dissemination plans for completed scoping review

A range of dissemination strategies will include sharing of review findings with local academic networks within the authors’ place of work and third level institution. Oral and poster presentations at national and international conferences such as U21 Research in Healthcare and the International Society for Anthrozoology (ISAZ) have already shown interest in the research question and future review findings. The International Family Nursing Association have accepted an abstract for oral presentation at their virtual conference, June 2021. Once the scoping review has been completed the authors will seek to publish in peer reviewed journals such as the Journal of Clinical Nursing and the International Journal of Social Research Methodology.

### Study status

This study is at Stage 2 – Identification of relevant studies. A review of reference management software has begun with a preliminary incorporation of search terms into the search engines cited.

## Discussion and conclusion

A scoping review protocol has been outlined and discussed in relation to the current literature and evidence available ( Daudt *et al.*, 2013; Levac *et al.*, 2010; Peters *et al.*, 2017 & Peters *et al.*, 2020). The value of implementing Animal Assisted Interventions into a children’s hospital requires careful planning, review of the evidence and risk assessment. The rationale for choosing a scoping review over other reviews is to map the scientific evidence for AAIs in children’s hospitals to inform any further research. From the initial search; carried out early 2020; mixed methods studies and systematic reviews on the topic of AAI were reported. However, the findings revealed a lack of consistency in research approaches and a need for a scoping review is warranted so that any further research can be planned appropriately to address any gaps in the scientific knowledge. The knowledge base of AAIs is still being created and evaluated. This scoping review will contribute to cataloguing the scientific evidence for AAIs in the children’s hospital context and inform a research project.

## Data availability

### Underlying data

No data are associated with this article.
