# Supplementary material for: Animal assisted interventions in the children's hospital: protocol for a scoping review
Source: HRB Open Res. 2021 May 14;3:74. Originally published 2020 Oct 5. [Version 2] doi: 10.12688/hrbopenres.13143.2 (PMC8136254; doi:10.12688/hrbopenres.13143.2)
Supplement: Peer review history

# Peer review: Reviewer response for version 2

**DOI:** 10.21956/hrbopenres.14479.r29455

**Published:** 2021-05-19

**Authors:** Tim Dornan, Gail Davison

**Affiliations:** 1 Centre for Medical Education, Queen's University Belfast, Belfast, UK; 2 Maastricht University, Maastricht, The Netherlands

The authors have addressed our concerns and made amendments accordingly. We have a few minor recommendations that can be addressed by proofing: 1. Change the spelling of ‘journaals’, 2. Update tables 1 & 2 (so that they appear chronologically), 3. In table 3, change ‘none’ to ‘no language restrictions’ and ‘no restrictions in methods’. We look forward to reading the completed review and wish the authors every success in researching this interesting topic.

Is the study design appropriate for the research question?

Partly

Is the rationale for, and objectives of, the study clearly described?

Partly

Are sufficient details of the methods provided to allow replication by others?

Partly

Are the datasets clearly presented in a useable and accessible format?

Not applicable

Reviewer Expertise:

Care of hospitalised children; scoping review; research methodology; medical education.

We confirm that we have read this submission and believe that we have an appropriate level of expertise to confirm that it is of an acceptable scientific standard.

# Peer review: Reviewer response for version 1

**DOI:** 10.21956/hrbopenres.14257.r28565

**Published:** 2021-01-22

**Authors:** Tim Dornan, Gail Davison

**Affiliations:** 1 Centre for Medical Education, Queen's University Belfast, Belfast, UK; 2 Maastricht University, Maastricht, The Netherlands

This review is written by Gail Davison, a senior paediatric trainee and PhD student, who has used scoping review to research the experience of sick children in hospital and Tim Dornan, former internist and education researcher. We share an interest in qualitative methodologies and, specifically those that give insight into sick people's experiences in order to improve healthcare.

 The protocol addresses an extremely important topic (non-pharmacological interventions to improve illness experience) which is all the more important because animal assisted activities are an interesting, unconventional type of therapy. The critical comments that follow are not at all intended to discourage the authors from conducting the review, but they are informed by GD’s experiences of scoping review, which might help the authors focus their work and have the greatest positive impact on children and parents.

 If this protocol does one thing, it illustrates the shortcomings of scoping reviews. It assumes that some circumscribed topic already exists, that there is a clearly defined thing called 'evidence' which bears on the topic, and that a gap in the evidence can be identified by assembling it. What this protocol illustrates is that, certainly regarding animal assisted activities, that is far from the case. As a result, the protocol is somewhat lacking in focus and clarity.

 In such an under-researched topic as this, there is a problem unanticipated by the Arksey and O'Malley framework: you first have to frame the topic in order to render it reviewable. Putting it more simply, if you simply round up the evidence, you will find it hard to decide what is and isn't evidence and will end up with a hotchpotch of information that can really only lead you to the conclusion that "more research is needed". We propose that the authors could do an extremely useful job by taking the vital step before that: clarify the field of inquiry. If you don't have any idea what you want to do for children and why, then, you will have no idea whether you should be interested in salivary cortisol levels or children missing their own pets. So, we suggest that a clearer definition of the field, which might clarify the possible impacts and their relative importance will make the selection and analysis of articles a lot easier and a lot more useful. The comments that follow turn those rather vague critical comments into more specific issues, which we hope will help the authors do what we suggest.

 Purpose

 Is this to gather evidence on what activities are being practised and how they influence children? Is it to explore why and how activities benefit children? Is it to understand under what conditions they are successful? Is it to examine which types of pets are most efficacious? Is it to improve experiences, or improve physiology and biochemistry? To show why we ask these questions, we draw the reviewers' attention to the first line of their review question. What do they mean by “science”, and what do they mean by “evidence base”? You will see that the answers to those questions will be completely different if you think salivary cortisol or the experience of being sick is more important. To give an example of this point, GD's scoping review uses children's verbatim accounts of their experiences as evidence and gives a careful justification for doing so. People who care about salivary cortisol would likely dismissive this evidence as fluffy and would find it hard to understand why we would dismiss salivary cortisol as irrelevant. Scoping reviewers need to come off the fence about this or, at least, acknowledge that there is a fence to come off. The reviewers show their own unclarity about this by flipping between the terms “activity" and "intervention". It might clarify their own minds if they used examples to clarify their own position. GD draws attention to the limited way this has been done in the protocol by drawing attention to a previous critical review of AAIs in children's hospitals, which the reviewers do not quote.

 For whom?

 Another important question is who the beneficiaries of AAIs should be. Is it children alone? Is it children and parents? Could it even be parents? We suggest that, in paediatric research, authors should clarify their position on whether children are autonomous individuals or part of a "unit" of which their parents are an inevitably dominant part. Whilst it would be possible to look at all 3 parties (children, parents and HCPs), this would broaden the focus and limit the amount that could be said about children.

 Risk

 We suggest that it would be a missed opportunity if the issue of risk were not addressed. The reviewers write about AAIs in intensive care units. Would a dog have to wear PPE in that setting? What would happen if it wanted to spend a penny? When it comes to implementing the findings of this review, those might be the single most important issues for implementors. Are virtual interactions in scope?

 Specific comments regarding the stages of scoping review:

 Stage 1

 We note that aims 1 and 2 are very little different from one another and would be appropriately placed with the research question. The heading ‘protocol’ could be removed. We suggest that terms like "children", "AAI", and "Children's Hospital setting" need to be defined, otherwise it will be hard to decide which articles are in or out of scope. Rather than seeking ‘scientific evidence’, we suggest either asking ‘what is known about animal assisted interventions for children and young people in children’s hospital settings?’ (leaving it open to unpublished evidence and grey literature) or ‘what is known about the scientific evidence base for …?’ (therefore, including research findings only). The sentence commencing ‘The population (P)…’ is unnecessary as it is self-explanatory.

 Stage 2

 The protocol is ambiguous regarding translation of non-English studies. The authors say that studies will be translated if they are valuable, but how can you know they're valuable if you haven't translated them? The protocol leaves it quite unclear how comprehensive the selection of articles will be. We suggest there is a real risk that the reviewers will be flooded by including grey literature, websites, and 14 databases with no limits on article types. The risk of being overwhelmed is made greater by the search strings, which are quite non-specific. We suggest a combination of MESH term searches and keyword searches (including compatible truncation, possibly locating keywords separated by 5-10 consecutive words).    

 Stage 3

 We suggest that the eligibility criteria and limits are insufficiently defined to make the process as straightforward as the primary author suggests. Since there is a risk of being overwhelmed by gathering all information, it might be helpful to give an example of what a highly relevant (gold standard) article might look like. Of course, these issues could be addressed by iterative focusing of the review, rather than addressing them at this stage, but there is a real danger that the reviewers might irretrievably lose focus before they ever became clear.

 Stages 4 and 5

 We have no comments about these.

 Stage 6

 This stage is described rather generally in terms of networking, PPI and dissemination of findings, whereas Arksey and O’Malley intended stage 6 to be a formal consultation exercise, for which research ethics and governance approvals would be needed. We suggest that the authors should be clearer about this.

 Discussion and conclusions

 As it stands, these are rather bland. We hope that our preceding comments will give the reviewers some ideas about strengths, limitations, suspected challenges, and ways of addressing these that might strengthen the discussion and conclusions

 We have some other small suggestions:

 The title uses the term “animal assisted activities", which is different from the abbreviation AAI. Certainly, the authors should be consistent. We think intervention would be the more appropriate term to use because an intervention is implemented for an intended outcome, where an activity could be purely for its own sake. The phrase "at home in Ireland” and primarily quoting Irish studies may be of primary interest to the authors, but this orientation towards the country in which research was done would not be so persuasive to editors and reviewers of influential journals.

 In summary, we appreciate that our comments may seem rather overwhelming. Actually, the message is rather simple. For your own sake, as well as for the sake of good scholarship, focus and be clear. Our comments are intended to help you do that.

Is the study design appropriate for the research question?

Partly

Is the rationale for, and objectives of, the study clearly described?

Partly

Are sufficient details of the methods provided to allow replication by others?

Partly

Are the datasets clearly presented in a useable and accessible format?

Not applicable

Reviewer Expertise:

Care of hospitalised children; scoping review; research methodology; medical education.

We confirm that we have read this submission and believe that we have an appropriate level of expertise to confirm that it is of an acceptable scientific standard, however we have significant reservations, as outlined above.

# Response

**Published:** 2021-04-22

**Authors:** Rachel Howe

**Affiliations:** University College Dublin, Ireland

Thank you Dr Dornan and Ms Davison for your time and valuable comments on our scoping review protocol. We have revised our protocol to reflect a more focused review question and take onboard your constructive criticism.  We want to review the scientific evidence base for Animal Assisted Interventions (AAI) in children and young people who are inpatients in the children’s hospital.  The authors acknowledge that ‘science’ and ‘evidence base’ are sometimes loosely and liberally applied.  In our protocol it means a map of the existing literature available through peer-reviewed journals irrespective of the quality.  We also want to probe the literature to determine; Why has AAI been used? Where? And how effective was it? Were there implementation considerations? Were there particular research methods utilised and recommendations for future research?

 Since the topic of AAI is relatively new in children’s healthcare; indeed it is only recognised in academic circles since the beginning of the 21st Century (DeMello, 2012); there is a need to scope out the research undertaken internationally and map the evidence using the bespoke data charting form designed.  The international scale of AAIs are important and hence the inclusion of non-English literature that meets the inclusion criteria.  The review team possesses international language skills to enable the translation of title and abstracts in the first screening process.  There is ambiguity in the international literature in relation to the definition and adaptation of the terms AAI (which is the umbrella term) animal assisted activities (AAA) and animal assisted therapy (AAT). Some studies use either concept or use them interchangeably. The subsequent scoping review will provide a clear definition of these terms from the literature. The scoping review will be undertaken to help frame and focus further research. The main focus of the research will be on the children and young people, their responses to and interactions with AAI. We acknowledge that parents/guardians and healthcare providers act as gatekeepers for minors with regards to consent and access for research.  Their input and opinions about the child or young person’s reaction to the AAI will be important and valuable data to consider later when planning the research methods.  Evaluating the responses of parents/guardians and healthcare providers of the AAIs would be another avenue of research which could be recommended for future research but not the focus of this study.  However, studies reporting views other than children will not be excluded from this review since very few studies report just the children’s views, and those that do, may not be reported separately.  

 The protocol stated that the research team includes a librarian (DS) with expert experience in carrying out searches using thesaurus tools and will provide assistance and support to the researcher carrying out the database searches. AAIs stem from the overarching discipline of Human-animal studies, which includes an array of multidisciplinarians and interdisciplinarians, justifying the need for searching so many databases.  The highly sensitive search strategy using boolean operators, both free-text and subject headings (including MeSH when available) for each database search will be reported to ensure transparency through a clear audit trail.  The keywords for each search string were clearly articulated in the protocol (Table 1.). The review team have also invested in Covidence for ease of screening and tracking decisions made on inclusion and exclusion criteria. 

 In relation to any potential risks; of bites or cross infection between animal and patients; there will be a need to ensure relevant policies and guidelines are implemented prior to AAI taking place.  Additional content on these risks has also been included within the updated review protocol. Therefore, hopefully these reservations will now be alleviated.

# Peer review: Reviewer response for version 1

**DOI:** 10.21956/hrbopenres.14257.r28554

**Published:** 2021-01-06

**Authors:** Mary Hughes

**Affiliations:** 1 Children’s Nursing, School of Nursing and Midwifery, Trinity College Dublin, Dublin, Ireland

Thank you for inviting me to review this very interesting manuscript. It is a novel topic and the rationale for the development of the protocol is presented in a clear and coherent manner. The development of the protocol follows a recognised framework and is based on the authors' understanding of the problem under investigation. 

 I suggest some minor changes to the manuscript, there are some errors in grammar and syntax which could be corrected with proof-reading. Some editing is required to reduce the duplication of information, and support the rationale for decisions made in the steps of the framework with reference to the literature.

 I look forward to reading the report on the scoping review of the literature.

Is the study design appropriate for the research question?

Yes

Is the rationale for, and objectives of, the study clearly described?

Yes

Are sufficient details of the methods provided to allow replication by others?

Yes

Are the datasets clearly presented in a useable and accessible format?

Not applicable

Reviewer Expertise:

Child Health and Wellbeing, Chronic Illness in Childhood, Children's nursing.

I confirm that I have read this submission and believe that I have an appropriate level of expertise to confirm that it is of an acceptable scientific standard.

# Response

**Published:** 2021-04-22

**Authors:** Rachel Howe

**Affiliations:** University College Dublin, Ireland

Thank you Dr Hughes for taking the time to review our scoping review protocol. We appreciate your positive feedback.  We will proof-read the subsequent protocol more carefully to reduce duplication of information and amend any grammar or syntax errors. Thank you again for your interest and constructive comments.
